# Oral Intake of Royal Jelly Has Protective Effects Against Tyrosine Kinase Inhibitor-Induced Toxicity in Patients with Renal Cell Carcinoma: A Randomized, Double-Blinded, Placebo-Controlled Trial

**DOI:** 10.3390/medicines6010002

**Published:** 2018-12-20

**Authors:** Kyohei Araki, Yasuyoshi Miyata, Kojiro Ohba, Yuichiro Nakamura, Tomohiro Matsuo, Yasushi Mochizuki, Hideki Sakai

**Affiliations:** Department of Urology, Nagasaki University Graduate School of Biomedical Sciences, 1-7-1 Sakamoto, Nagasaki, Nagasaki 852-8501, Japan; k-araki205@cameo.plala.or.jp (K.A.); ohba-k@nagasaki-u.ac.jp (K.O.); yn1238056@yahoo.co.jp (Y.N.); tomozo1228@hotmail.com (T.M.); mochi@nagasaki-u.ac.jp (Y.M.); hsakai@nagasaki-u.ac.jp (H.S.)

**Keywords:** royal jelly, adverse events, tyrosine kinase inhibitors, renal cell carcinoma, double-blinded, randomized clinical trial

## Abstract

**Background:** Although tyrosine kinase inhibitors (TKIs) are still recommended as the standard therapy in renal cell carcinoma (RCC), the high frequency of adverse events is a weakness of this therapy. Because royal jelly (RJ) possesses anti-inflammatory and antioxidant properties, we assessed its protective effects on TKI-induced toxicities in RCC patients. **Methods:** We enrolled 33 patients with advanced RCC who were assigned to start TKI therapy in combination with a randomized, double-blinded, placebo-controlled RJ trial consisting of a placebo group with 17 subjects and an RJ group with 16 subjects. **Results:** Fatigue and anorexia frequencies in the RJ group were significantly lower than in the placebo group (*p* = 0.003 and 0.015, respectively). A statistically significant correlation between RJ and fatigue or anorexia was detected in sunitinib-treated patients. The dose reduction- or discontinuation-free periods were significantly longer (*p* = 0.013) in the RJ group than in the placebo group. Furthermore, similar observations were made in sunitinib-treated patients (*p* = 0.016). **Conclusions:** Our clinical trial showed that RJ exerted protective effects against TKI-induced fatigue and anorexia and lowered TKI dose reduction or discontinuation. Hence, RJ is beneficial for maintaining the quality of life and medication compliance in TKI-treated RCC patients.

## 1. Introduction

Renal cell carcinoma (RCC) is one of the most common urological cancers, and its incidence has continuously increased over the past few decades [1]. Although a nephrectomy is usually performed for organ-confined RCC, additional systematic therapy is the standard treatment strategy in patients with metastatic RCC. Currently, new treatment options including immune check-point inhibitors are being developed; however, a molecularly targeted therapy using tyrosine kinase inhibitors (TKIs) is still the recommended standard therapy for these patients [2,3,4]. In addition, molecularly targeted therapies are often used as neoadjuvant therapy for cytoreductive nephrectomy [5], and clinical trials of combination therapies including molecularly targeted therapeutics and other anticancer agents are currently in progress [6,7,8]. On the other hand, a major limitation of molecularly targeted therapies is the relatively high frequency of adverse events (AEs) with occasionally severe effects [9,10]. Therefore, the management of drug-induced AEs is critical for maintaining the quality of life during treatment and continuous therapy in these patients.

Sunitinib and pazopanib are approved as first-line therapy for patients with RCC, especially in favorable- or intermediate-risk clear cell RCC [2]. In addition, these TKIs are often used in real-world patients with non-clear cell RCC [11]. On the other hand, axitinib and sorafenib are also used in some patients with metastatic RCC [12,13]. Typical AEs linked to these TKIs include various symptoms such as oral mucositis, hand–foot syndrome, hypertension, fatigue, and gastrointestinal events [14,15,16,17,18]. In addition, functional disorders of the kidneys, liver, and thyroid are often associated with TKIs [14,15,17]. The occurrence and progression of these AEs are suggested to be mediated by complex mechanisms that involve inflammation, oxidative stress, and the immune system [19,20,21]. Based on these findings, we hypothesized that controlling these biological factors may suppress TKI-induced adverse events.

Royal jelly (RJ) is secreted by the hypopharyngeal and mandibular glands of worker honeybees of *Apis mellifera*, and is the exclusive food for the queen honeybee and larvae. The most important biological effects of RJ are its anti-inflammatory and antioxidative activities, and its ability to exert some control over the immune system [22,23,24]. These RJ-related activities are predicted to be beneficial in the protection against anticancer agent-induced adverse events involving inflammation, oxidative stress, and immune system dysfunction [25,26]. In fact, the protective efficacy of RJ against anticancer therapy associated toxic effects, such as oral mucositis, intestinal damage, and nephro- and hepato-toxicities, has been demonstrated in animal models with malignancies [27,28,29,30].

Thus, in recent decades, several studies have examined the biological effects of RJ on cancer cell lines and corresponding animal models. However, despite several clinical trials, not many studies have reported the clinical benefits and limitations of RJ administration in cancer patients [31,32,33,34,35]. Specifically, there are no reports about the effects of RJ on the adverse events caused by molecularly targeted therapies in RCC patients. Therefore, the aim of this study was to assess the protective efficacy of RJ against TKI-induced toxicities in patients with RCC. 

## 2. Methods

### 2.1. Patients 

This study protocol was approved by the Human Ethics Review Committee of Nagasaki University Hospital (Nagasaki, Japan; No. 15102604-2), and it was registered as UMIN000020152. In addition, this trial was conducted according to the Declaration of Helsinki. Patients provided written informed consent to participate in all aspects of the study. Patients with RCC who had been assigned to start TKIs were enrolled by the Nagasaki University Hospital. Eligibility criteria of this trial were age >20 years, Eastern Cooperative Oncology Group performance status (ECOG PS) 0 or 1, and no honey allergy. 

### 2.2. Study Design

This is a randomized, double-blinded, placebo-controlled clinical trial. Patients were assigned to two groups, namely the placebo and RJ groups, at a ratio of 1:1 using computer-generated random numbers. The patient selection process was performed by independent non-medical staff at our hospital who had no information on the aim of this trial, and the process was also hidden from the patients and urologists who provided treatment until the end of all analyses. 

RJ and the placebo were provided by the Yamada Agriculture Center Inc (Okayama, Japan). RJ and the placebo were prepared as capsules containing 900 mg RJ and starch, respectively, that share the same taste, smell, size, shape, and color. Capsules were orally administered four times per day for three months.

### 2.3. Protocol

All subjects received medical examinations and were checked for clinical symptoms every two weeks. Blood samples were collected simultaneously every two weeks and subjected to laboratory analysis routinely performed in patients treated with TKIs. The initial starting dose of sunitinib, pazopanib, axitinib, and sunitinib was 50 mg/day, 800 mg/day, 10 mg/day, and 800 mg/day, respectively, but if an intolerable AE was observed, the dose was decreased to 37.5 mg/day, 400–600 mg/day, 5 mg/day, and 400 mg/day, respectively. In addition, for the sunitinib regimen, if the dose reduction was necessary, all affected patients were treated with an alternative every other day [36]. Furthermore, TKI administration was stopped if intolerable AEs persisted or high-grade abnormalities in the blood analysis were observed. The primary outcome was the frequency and severity of AEs caused by TKIs in patients with RCC, and the secondary outcome was the sustained period of the initial TKI regimen. 

Toxicities were graded according to the National Cancer Institute Common Terminology Criteria for Adverse Events, version 5.0. In this study, toxicities classified as grade 3 and 4, or identified as the cause for discontinued TKI administration, were judged as severe AEs. 

### 2.4. Statistical Analyses

Results are expressed as the mean and standard deviation (SD) for normally distributed data and the median and interquartile range (IQR) for non-normally distributed data. The Student’s t-test or Mann–Whitney U test was used to compare continuous variables and Scheffé’s method was used for multiple comparisons. The sustained periods of initial dosage were derived from Kaplan–Meier curves, and statistical significance was analyzed using the log-rank test. Values with *p* ˂ 0.05 were considered statistically significant. Statistical analyses were carried out using StatView for Windows v.5.0 software (Abacus Concept, Berkeley, CA, USA).

## 3. Results

All subjects of the placebo and RJ group consumed the respective capsules for three months or until disease progression. In addition, we confirmed that the compliance rate was >95% in both groups and no patient experienced any side effects including allergy due to the trial capsules. 

### 3.1. Patient Background 

Thirty-three patients with RCC were enrolled in this study. The pathological features and basic characteristics of our study population at baseline are presented in Table 1. 

The statistical analysis indicated that the baseline parameters did not significantly differ between the placebo and the RJ groups. As shown in Table 1, among the 33 patients, 21, 8, and 3 patients were treated with sunitinib, pazopanib, and axitinib, respectively, whereas only one patient was treated with sorafenib. The patients were further divided into two subgroups, namely the sunitinib and the other TKI group, because 63.5% of the patients were treated with sunitinib. The clinicopathological features and basic characteristics of each subgroup are listed in Table 2. 

In the other TKI group, the mean age of the RJ group was significantly higher (*p* = 0.013) compared to that of the RJ group. However, the remaining parameters did not significantly vary between these two groups. 

### 3.2. Adverse Events 

In the study population (*n* = 33), the most common AE was hypertension (*n* = 23; 69.7%) followed by fatigue (*n* = 20; 60.6%), and anorexia (*n* = 18; 60.0%) and hand–foot syndrome (*n* = 18; 60.0%). Furthermore, anorexia (*n* = 4; 12.1%) was the most common severe adverse event. 

As shown in Table 3, the frequencies and severities of fatigue and anorexia were significantly lower in the RJ group than those in the placebo group (*p* = 0.003 and 0.015, respectively). The digestive symptoms varied similarly, but the difference between the RJ and placebo group was not statistically significant (*p* = 0.077). In addition, none of the other symptoms varied significantly, including hypertension. However, when the same analysis was performed separately on the subgroups of patients treated with either sunitinib or other TKIs, such significant differences in fatigue and anorexia differed significantly between the RJ and placebo groups in patients treated with sunitinib (*p* = 0.040 and 0.038, respectively), but not in those treated with other TKIs (*p* = 0.065 and 0.343, respectively). The frequencies of the remaining symptoms were similar between the two groups regardless of the TKI regimen. 

Table 4 shows the laboratory blood test results in relation to the trial capsule treatment groups. The frequency of patients with normal anemia values was higher in the RJ group (87.5%) than in the placebo group (58.8%); however, the difference was not statistically significant (*p* = 0.162). When the same analysis was performed with the data of the sunitinib-treated patients, no significant difference was detected (*p* = 0.117). Overall, none of the laboratory blood test results varied significantly between the placebo and the RJ group (Table 4).

### 3.3. Dose Reduction or Discontinuation of Tyrosine Kinase Inhibitors

**Figure 1:**
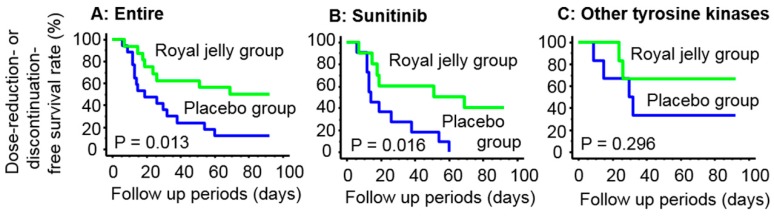
Kaplan–Meier survival curves of the dose-reduction- or discontinuation-free survival rate in the placebo and RJ groups. Dose-reduction- or discontinuation-free survival was better in the RJ group than in the placebo group for patients treated with any TKI (**A**) and sunitinib (**B**) (*p* = 0.127 and 0.016, respectively). However, this difference was not observed in patients treated with other TKIs (**C**).

In our study population, 23 of the 33 patients (69.7%) required a dose reduction or discontinuation of the TKI regimen due to severe AEs and disease progression within three months of TKI treatment initiation. The frequency of dose reduction or discontinuation was significantly lower (*p* = 0.017) in the RJ group (5/16 = 50.0%) than in the placebo group (15/17 = 88.2%). The Kaplan–Meier survival curves for dose-reduction- or discontinuation-free survival rates in the placebo and RJ group are shown in Figure 1A. The free-periods were significantly longer (*p* = 0.013) in the RJ group than in the placebo group. Furthermore, a similar result was observed in patients treated with sunitinib (*p* = 0.016; Figure 1B). However, although the dose-reduction- or discontinuation-free survival rates appeared to be better in the RJ group than in the placebo group among patients treated with TKIs other than sunitinib, the difference between these subgroups was not significant (*p* = 0.296; Figure 1C).

## 4. Discussion

In this study, we investigated the preventive effects of prophylactic RJ consumption on AEs associated with TKI-induced toxicities because the appropriate management of these AEs is critical for maintaining quality of life in RCC patients treated with TKIs. Various harmful symptoms and abnormal observations are known as adverse events of TKIs [12,14,15,16,17,18]. Among these chemotherapy-induced symptoms, such as oral mucositis, anorexia, digestive symptoms, fatigue, and damage of the kidneys and liver, few have been shown to be suppressed by RJ administration in animal experiments and clinical trials [27,28,29,30,31,32,33,34,37]. In addition, it is known that RJ improves protection against hypertension, hand–foot syndrome, and bone marrow suppression, which are representative adverse events associated with TKIs [38,39]. However, we found that among these harmful symptoms, RJ administration was not significantly associated with the frequency and severity of hypertension, hand–foot syndrome, oral mucositis, dysgeusia, and kidney or liver damage. Importantly, we demonstrated that fatigue and anorexia in the RJ group were significantly mild compared to that in the placebo group. Interestingly, RJ has been shown to exert anti-fatigue effects in an animal experiment that investigated fatigue-related parameters such as serum lactate, serum ammonia, and muscle glycogen after swimming in mice treated with RJ [40]. The study found that these parameters were improved by RJ intake [40]. However, we should note that this fatigue was not induced by anticancer therapies such as TKIs. However, there is a report that processed honey and RJ ameliorated cancer-related fatigue in a double-blinded, randomized clinical trial [33]. Specifically, the visual analogue fatigue scale and fatigue severity score in the test article group (processed honey and RJ for four weeks; *n* = 26) were significantly lower than those in the control group (pure honey for four weeks; *n* = 26) [33]. We believe that the present study supports this previous result. However, the previous study had some limitations; for example, the study population included six different types of cancer (breast, gastric, esophageal, colon, rectal, and prostate cancer). Although there was no statistical difference between the composition of the study and control group, the patients had to be treated with four different methods (hormonal therapy, chemotherapy, chemoradiation, and radiotherapy). Furthermore, as discussed by the authors, the short duration of intervention (four weeks) was another limitation [33]. Thus, the present study is the first report on the association between RJ and cancer-related fatigue in RCC patients treated with TKIs. Fatigue is widely recognized as a highly common adverse event in TKI-treated patients with metastatic RCC (sunitinib, 55%; pazopanib, 27%; axitinib, 9%; and sorafenib; 8%) [12]. In addition, it was observed that only 4% of these patients received pharmacologic treatment for fatigue, whereas 72% of hypertension was treated [12]. Moreover, there are only a few effective treatment options for fatigue caused by TKIs. Hence, our observation that RJ may significantly suppress TKI-induced fatigue is an important finding for TKI-based treatment strategies.

In addition to fatigue, our results demonstrated that RJ suppressed the frequency and severity of anorexia. To our knowledge, this is the first report about the preventive effect of RJ on anorexia in RCC patients. In addition, there is no information on the relationship between RJ and anorexia in patients with malignancies. In general, anorexia in cancer patients is caused by complex interactions involving various factors, such as nausea, constipation, pain, depression, and hypothyroidism [41]. In addition, it is recommended that anorexia treatment should include drugs that target the following conditions: nutritional disorders, muscle catabolism, anemia, and fatigue [42]. Furthermore, it was reported that inflammatory status, oxidative stress, and immunosuppression are important targets for anorexia treatment. From our results, we cannot describe the interactions induced by RJ to exert the preventive effects against anorexia. However, we speculate that there are multiple beneficial effects of RJ on various TKI-induced adverse events including fatigue, digestive symptoms, anemia, and nephro- and hepato-toxicities that contribute to this finding, although RJ is not significantly associated with the prevention of these events. Furthermore, RJ has been reported to possess anti-inflammatory and antioxidative activities, and act as a significant regulator of immune conditions caused by anticancer therapies [35]. We agree with the opinion that the clinical management of anorexia requires a multidisciplinary and multi-pharmacological approach [42]. As useful information for TKI-treatment management, RJ intake is beneficial to prevent anorexia in cancer patients treated with sunitinib. 

Our results showed that RJ had no significant effect on oral mucositis. However, a preliminary study designed as a randomized, single-blinded (physician-blinded) trial showed that prophylactic RJ use led to a significant reduction in head and neck cancer patients. However, the study population was small (the RJ and control groups had seven and six patients, respectively) [32]. On the other hand, other investigators also reported that RJ improved symptoms of oral mucositis and shortened the healing time in 103 patients [31]. However, in these two studies, patients were treated with a combination of chemotherapy and radiotherapy. We believe that RJ may prevent the oral mucositis caused by chemotherapy and/or radiotherapy, but not by TKIs.

Our results showed that there was no significant relationship between RJ consumption and the blood test results. Several in vivo studies demonstrated RJ administration led to the protection of various organ functions [35]. For example, RJ strongly suppressed clinical and pathological aggravation of the liver, kidneys, and testis in experimental animals treated with cisplatin [29,30,37]. On the other hand, in a human trial, the serum levels of creatinine and urea were significantly increased during the first and second cycle of cisplatin-based chemotherapy in 32 cancer patients, but the increase in kidney function-related parameters was suppressed by RJ administration. However, the nephroprotective activity was not statistically significant [34]. Our results are similar to those reported in this study, and we assume that differences such as species, dosage, and period of RJ treatment as well as physiological characteristics of the kidneys are causes for the different observations in animal models and cancer patients. 

Our study design has several limitations that restrict the conclusions about the preventive effects of RJ on AEs caused by TKIs in patients with RCC. First, the trial had a relatively small number of patients in each group. However, we emphasize that this trial is a preliminary study on the RJ consumption by patients with malignancy treated with TKIs. In addition, the number of patients in the present study appears to be relatively similar to that reported in previous clinical trials on RJ administration [32,33]. Second, the RJ capsules were provided by a company that sells supplements made from honey. To ensure that the study is not biased, we performed this trial as a double-blinded, randomized study, and the administration, data collection, and analysis were done by a third party approved by the Human Ethics Review Committee. Therefore, our results were not affected by the company and we did not receive any financial support for the publication of this manuscript. Finally, our study design cannot identify the active substance causing the beneficial functions of RJ. Although RJ contains mostly sugars, lipids, amino acids, and vitamins, 10-hydroxy-2-decenoic acid (10-HDA), royalisin, and apisin are known as major components with pharmacological activities [43,44,45]. We assume that some of these RJ-specific components may cause the observed effects. Further detailed analyses are necessary to identify the active ingredients. 

In conclusion, our results demonstrated that prophylactic RJ intake is effective for the prevention and suppression of sunitinib-induced fatigue and anorexia. In addition, RJ did not affect treatment safety and compliance. Specifically, we found that the risk of TKI dose reduction or discontinuation was significantly lower in the RJ group than in the placebo group. Sunitinib remains a recommended standard agent used singly or in combination with immune therapy, low molecular weight heparin, and vaccines for the treatment of metastatic RCC [6,7]. Therefore, we believe that our results are important to improve the treatment strategies for RCC. On the other hand, we emphasize that further detailed clinical studies with more participants are needed to determine whether RJ intake prevents TKI-induced AEs in cancer patients.

## Figures and Tables

**Table 1:** Clinicopathological features and basic characteristics.

Variables	Entire (*n* = 33)	Placebo (*n* = 17)	Royal Jelly (*n* = 16)	*p* Value
Age				0.101
Mean ± SD, years	67.6 ± 6.6	65.8 ± 8.8	69.6 ± 5.9	
Gender; *n* (%)				0.909
Male/Female	23/10 (30.3)	12/5 (29.4)	11/5 (31.3)	
Performance Status				0.598
0/1	16/17 (51.5)	9/8 (47.1)	7/9 (56.3)	
Pathological Type				0.446
Conventional	29 (87.9)	16 (94.1)	13 (81.3)	
Fuhrman Grade				0.425
1 or 2/3 or 4	6/27 (81.8)	2/15 (88.2)	4/12 (75.0)	
pT stage				0.201
1 or 2/3 or 4	9/24 (72.7)	3/14 (82.4)	6/10 (62.5)	
Lymph Node Metastasis				0.881
Presence	19 (57.6)	10 (58.8)	9 (56.3)	
Distant Metastasis				0.325
Presence	27 (81.8)	15 (88.2)	12 (75.0)	
Neo-Adjuvant Setting				0.965
Yes	2 (6.1)	1 (5.9)	1 (6.3)	
Past Therapy Used TKI				0.948
Presence	4 (12.1)	2 (11.8)	2 (12.5)	
TKIs				0.539
Sunitinib	21 (63.6)	11 (64.7)	10 (62.5)	
Pazopanib	7 (21.2)	3 (17.6)	4 (25.0)	
Axitinib	4 (12.1)	3 (17.6)	1 (6.3)	
Sorafenib	1 (3.0)	0 (0.0)	1 (6.3)	

TKIs = tyrosine kinase inhibitors. SD = standard deviation.

**Table 2:** Clinicopathological features and basic characteristics according to used agents.

Variables	Placebo	Royal Jelly	*p* Value
Sunitinib	*n* = 11	*n* =10	
Age (mean ± SD), years	66.5 ± 8.3	67.8 ± 5.8	0.673
Gender; Male/Female, *n* (%)	6/5 (45.5)	8/2 (20.0)	0.217
Performance Status; 0/1	7/4 (36.4)	6/4 (40.0)	0.864
Pathological Type; Conventional	11 (100.0)	8 (80.0)	0.297
Fuhrman grade; 2/3+4	1/10 (90.9)	2/8 (80.0)	0.476
pT stage; 1+2/3+4	2/9 (81.8)	3/7 (70.0)	0.525
Lymph Node Metastasis; Presence	7 (63.6)	6 (60.0)	0.864
Distant Metastasis; Presence	9 (81.8)	8 (80.0)	0.916
Neo-Adjuvant Setting; Yes	1 (9.1)	0 (0.0)	0.329
Past Therapy Used TKI; Presence	1 (9.1)	0 (0.0)	0.329
Others	*n* = 6	*n* = 6	
Age (mean ± SD); years	64.5 ± 3.8	72.5 ± 5.3	0.013
Gender; Male / Female	6/0 (0.0)	3/3 (50.0)	0.182
Performance Status; 0/1	2/4 (66.7)	1/5 (83.3)	0.505
Pathological Type; Conventional	5 (83.3)	5 (83.3)	0.999
Fuhrman grade; 2/3+4	1/5 (83.3)	3/3 (50.0)	0.221
pT Stage; 1+2/3+4	1/5 (83.3)	2/4 (66.7)	0.501
LN Metastasis; Presence	3 (50.0)	3 (50.0)	0.999
Distant Metastasis; Presence	6 (100.0)	4 (66.7)	0.121
Neo-Adjuvant Setting; Yes	0 (0.0)	1 (16.7)	0.296
Past Therapy Used TKI; Presence	1 (16.7)	2 (33.3)	0.505

**Table 3:** Relationships between royal jelly intake and clinical symptoms.

Adverse Events	Entire		Sunitinib		Others	
	Placebo (*n* = 17)	RJ (*n* = 16)	Placebo (*n* = 11)	RJ (*n* = 10)	Placebo (*n* = 6)	RJ (*n* = 6)
**Hypertension**						
Nothing	6 (35.3)	4 (25.0)	5 (45.5)	3 (30.0)	1 (16.7)	1 (16.7)
Mild	10 (58.8)	12 (75.0)	6 (54.5)	7 (70.0)	4 (66.7)	5 (83.3)
Severe	1 (5.9)	0 (0.0)	0 (0.0)	0 (0.0)	1 (16.7)	0 (0.0)
*p* value	0.460		0.466		0.574	
**Fatigue**						
Nothing	2 (11.8)	11 (68.8)	1 (9.1)	6 (60.0)	1 (16.7)	5 (83.3)
Mild	13 (76.5)	5 (31.3)	9 (81.8)	4 (40.0)	4 (66.7)	1 (16.7)
Severe	2 (11.8)	0 (0.0)	1 (9.1)	0 (0.0)	1 (16.7)	0 (0.0)
*p* value	0.003		0.040		0.065	
**Anorexia**						
Nothing	4 (23.5)	11 (68.8)	3 (27.3)	8 (80.0)	1 (16.7)	3 (50.0)
Mild	9 (52.9)	5 (31.3)	5 (45.5)	2 (20.0)	4 (66.7)	3 (50.0)
Severe	4 (23.5)	0 (0.0)	3 (27.3)	0 (0.0)	1 (16.7)	0 (0.0)
*p* value	0.015		0.038		0.343	
**Digestive Symptoms**						
Nothing	6 (35.3)	11 (68.8)	3 (27.3)	7 (70.0)	3 (50.0)	4 (66.7)
Mild	8 (47.1)	5 (31.3)	6 (54.5)	3 (30.0)	2 (33.3)	2 (33.3)
Severe	3 (17.6)	0 (0.0)	2 (18.2)	0 (0.0)	1 (16.7)	0 (0.0)
*p* value	0.077		0.102		0.565	
**Dysgeusia**						
Nothing	7 (41.2)	9 (56.3)	5 (45.5)	5 (50.0)	2 (33.3)	4 (66.7)
Mild	9 (52.9)	7 (43.8)	5 (45.5)	5 (50.0)	4 (66.7)	2 (33.3)
Severe	1 (5.9)	0 (0.0)	1 (9.1)	0 (0.0)	0 (0.0)	0 (0.0)
*p* value	0.479		0.621		0.248	
**Hand–Foot Syndrome**						
Nothing	7 (41.2)	8 (50.0)	3 (27.3)	4 (40.0)	4 (66.7)	4 (66.7)
Mild	9 (52.9)	8 (50.0)	8 (72.7))	6 (60.0)	1 (16.7)	2 (33.3)
Severe	1 (5.9)	0 (0.0)	0 (0.0)	0 (0.0)	1 (16.7)	0 (0.0)
*p* value	0.578		0.537		0.513	
**Oral Mucositis**						
Nothing	9 (52.9)	11 (68.8)	7 (63.6)	7 (70.0)	2 (33.3)	4 (66.7)
Mild	8 (47.1)	5 (31.3)	4 (36.4)	3 (30.0)	4 (66.7)	2 (33.3)
Severe	0 (0.0)	0 (0.0)	0 (0.0)	0 (0.0)	0 (0.0)	0 (0.0)
*p* value	0.353		0.757		0.248	

RJ = royal jelly.

**Table 4:** Relationships between royal jelly intake and results of blood examinations.

Adverse Events	Entire		Sunitinib		Others	
	Placebo (*n* = 17)	RJ (*n* = 16)	Placebo (*n* = 11)	RJ (*n* = 10)	Placebo (*n* = 6)	RJ (*n* = 6)
**Leukopenia**						
Nothing	11 (35.3)	11 (25.0)	5 (45.5)	6 (60.0)	6 (100.0)	5 (83.3)
Low	5 (58.8)	5 (75.0)	5 (45.5)	4 (40.0)	0 (0.0)	1 (16.7)
High	1 (5.9)	0 (0.0)	1 (9.1)	0 (0.0)	0 (0.0)	0 (0.0)
*p* value	0.616		0.561		0.296	
**Anemia**						
Nothing	10 (58.8)	14 (87.5)	4 (36.4)	8 (80.0)	6 (100.0)	6 (100.0)
Mild	6 (35.3)	2 (12.5)	4 (36.4)	2 (20.0)	0 (0.0)	0 (0.0)
Severe	1 (5.9)	0 (0.0)	1 (9.1)	0 (0.0)	0 (0.0)	0 (0.0)
*p* value	0.162		0.117		> 0.999	
**Platelets**						
Nothing	5 (29.4)	9 (56.3)	2 (18.2)	5 (50.0)	3 (50.0)	4 (66.7)
Mild	8 (47.1)	4 (25.0)	6 (54.5)	3 (30.0)	2 (33.3)	1 (16.7)
Severe	4 (23.5)	3 (18.8)	3 (27.3)	2 (20.0)	1 (16.7)	1 (16.7)
*p* value	0.274		0.295		0.788	
**Renal Dysfunction**						
Nothing	8 (47.1)	10 (62.5)	5 (45.5)	6 (60.0)	3 (50.0)	4 (66.7)
Mild	9 (52.9)	6 (37.5)	6 (54.5)	4 (40.0)	3 (50.0)	2 (33.3)
Severe	0 (0.0)	0 (0.0)	0 (0.0)	0 (0.0)	0 (0.0)	0 (0.0)
*p* value	0.373		0.505		0.558	
**Liver Dysfunction**						
Nothing	12 (70.6)	14 (87.5)	7 (63.6)	9 (90.0)	5 (83.3)	5 (83.3)
Mild	4 (23.5)	1 (6.3)	4 (36.4)	1 (10.0)	0 (0.0)	0 (0.0)
Severe	1 (5.9)	1 (6.3)	0 (0.0)	0 (0.0)	1 (16.7)	1 (16.7)
*p* value	0.382		0.621		0.999	
**Thyroid Abnormality**						
Nothing	9 (52.9)	10 (62.5)	7 (63.6)	7 (70.0)	2 (33.3)	3 (50.0)
Mild	7 (41.2)	6 (37.5)	4 (36.4)	3 (30.0)	3 (50.0)	3 (50.0)
Severe	1 (5.9)	0 (0.0)	0 (0.0)	0 (0.0)	1 (16.7)	0 (0.0)
*P* value	0.577		0.757		0.549	
