# Correction: Oral Intake of Royal Jelly Has Protective Effects against Tyrosine Kinase Inhibitor-Induced Toxicity in Patients with Renal Cell Carcinoma: A Randomized, Double-Blinded, Placebo-Controlled Trial. *Medicines*, 2019, *6*, 2

**DOI:** 10.3390/medicines7110071

**Published:** 2020-11-19

**Authors:** Kyohei Araki, Yasuyoshi Miyata, Kojiro Ohba, Yuichiro Nakamura, Tomohiro Matsuo, Yasushi Mochizuki, Hideki Sakai

**Affiliations:** Department of Urology, Nagasaki University Graduate School of Biomedical Sciences, 1-7-1 Sakamoto, Nagasaki, Nagasaki 852-8501, Japan; k-araki205@cameo.plala.or.jp (K.A.); ohba-k@nagasaki-u.ac.jp (K.O.); yn1238056@yahoo.co.jp (Y.N.); tomozo1228@hotmail.com (T.M.); mochi@nagasaki-u.ac.jp (Y.M.); hsakai@nagasaki-u.ac.jp (H.S.)

We, the authors, wish to make the following correction to our published paper [1]

Section 2.2. Study Design

RJ and the placebo were prepared as capsules containing 900 mg RJ and starch, respectively, that share the same taste, smell, size, shape, and color. Capsules were orally administered four times per day for three months.

Should be replaced with

RJ and the placebo were prepared as capsules containing 800 mg RJ and starch, respectively, that share the same taste, smell, size, shape, and color. Capsules were orally administered three times per day for three months.

The reason why we, the authors, want to make a change is that this manuscript states that a total of 3600 mg (900 mg/dose, four times a day) of royal jelly (RJ) was administered. However, in fact, a total of 2400 mg of RJ (800 mg/dose, three times a day) was administered.

This error occurred because we mistakenly used information from another study, which we were planning while writing this manuscript.

In the other study, the dose is a total of 3600 mg (900 mg/dose, 4 times a day).

We are certain that the dose administered in this study was 800 mg/dose, three times a day.

We explain the reasons below:

# The informed consent form states that a subject receives oral administration of RJ three times a day. All participants understood the administration method before they provided consent.

# Both the study protocol and a disclosure form to the UMIN also state that the dosage is three times a day. All the researchers complied with this condition.

# During the study period, no information related to four doses a day was available.

# The stated 900 mg capsule has not been manufactured.

# The fact that the capsule administered to subjects (Royal Jelly King) contains 800 mg of RJ has been made public on various types of media and through the website in “Yamada Bee Farm”.

# We have confirmed, with all subjects or their family members, that the subjects took the capsule three times a day.

# We confirmed with each subject at every appointment that they were taking the capsule three times a day.

# At the beginning of the study, subjects received 300 capsules in total, which was adequate for three months. Therefore, the subjects could not have taken the capsule four times a day.

The authors would like to apologize for any inconvenience caused to readers by these changes. The changes do not affect the scientific results. No further amendment is required for other parts of the study methods or the results.

Furthermore, we anticipate no research ethics issues, as we complied with the dose and administration methods approved by the ethics committee. The original manuscript will remain online on the article webpage, with reference to this Correction.
